# Study on the Bacterial Communities of the Biofilms on Titanium, Aluminum, and Copper Alloys at 5,772 m Undersea in Yap Trench

**DOI:** 10.3389/fmicb.2022.831984

**Published:** 2022-03-18

**Authors:** Xiaofan Zhai, Wei Cao, Yimeng Zhang, Peng Ju, Juna Chen, Jizhou Duan, Chengjun Sun

**Affiliations:** ^1^Key Laboratory of Marine Eco-Environmental Science and Technology, Marine Bioresource and Environment Research Center, First Institute of Oceanography, Ministry of Natural Resources, Qingdao, China; ^2^CAS Key Laboratory of Marine Environmental Corrosion and Bio-Fouling, Institute of Oceanology, Chinese Academy of Sciences, Qingdao, China; ^3^Open Studio for Marine Corrosion and Protection, Pilot National Laboratory for Marine Science and Technology (Qingdao), Qingdao, China; ^4^Center for Ocean Mega-Science, Chinese Academy of Sciences, Qingdao, China; ^5^Navy Submarine Academy, Qingdao, China

**Keywords:** microbial communities, biofilm, metal alloys, hadal environment, Yap Trench

## Abstract

Biofilms formed on metal surfaces strongly affect metallic instruments serving in marine environments. However, due to sampling difficulty, less has been known about the bacterial communities of the biofilm on metallic surfaces in hadal environments, so the failure process of these deep-sea metallic instruments influenced by microbial communities could be hardly predicted. In this research, seven alloys, including titanium, aluminum, and copper alloys, were exposed in Yap Trench hadal environment for 1 year. Thus, the communities of the biofilms formed on metallic surfaces at 5,772 m undersea in Yap Trench were initially reported in previous studies. Then, 16S rRNA gene sequencing was performed to visualize the *in situ* bacterial communities of the biofilms formed on titanium, aluminum, and copper alloys at 5,772 m undersea in Yap Trench. It was found that Proteobacteria was the dominant phylum in all samples, but distinct genera were discovered on various alloys. The titanium alloy provided a suitable substrate for a mutualistic symbiotic biofilm with abundant bacterial richness. Aluminum alloys without copper components showed the least bacterial richness and formed a cold-adapted and oligotrophic-adapted biofilm containing the genera *Sulfurimonas* and *PS1 Clade*, while copper-present alloys showed relatively high bacterial richness with copper-resistant or even copper-utilizing biofilms constituting the genera *Stenotrophomonas*, *Burkholderia-Caballeronia-Paraburkholderia*, and *Achromobacter* on the surfaces. Furthermore, among all the element components contained in alloys investigated in this research, copper element showed the strongest influences on the composition and function of microbial communities in the biofilms formed on various metallic surfaces.

## Introduction

The hadal biosphere at the deep-sea environment in Yap Trench has been less studied and poorly explored. Until the recent years, microorganisms started to come to light owing to technical development (Li L. et al., 2020; Zhang et al., 2021). Microbial diversity in seawater and sediments was initially reported by the researchers. The microbial community composition in seawater was found to be dominated by Gammaproteobacteria with heterotrophic processes as the most common metabolisms (Zhang et al., 2018), whereas in the sediments, the microbial populations had fluctuating distributions and chemolithoautotrophic metabolic processes dominated by Proteobacteria and Thaumarchaeota (Fu et al., 2020). The studies showed that, in this dark realm, unique and highly adapted microbial communities have formed. Especially the detection/enrichment of genes involved in stress response and metal resistance in the seawater and sediment of the Yap Trench suggested special adaptation strategies of the hadal microorganisms toward high pressure and/or nutrient availability, while the enrichment of metal resistance genes might be a hypothesized characteristic of the hadal seawater microbial communities (Zhang et al., 2018). Besides that, a typical “V-shape” topography, and frequent sediment collapses on trench walls, high total organic carbon (TOC%) and total nitrogen (TN%) were found in this environment, especially in the core sediments with distinct microbial populations of *Proteobacteria* and *Thaumarchaeota* (Fu et al., 2020). However, the details of the species and functions are still unknown.

At present, further studies on hadal environments are highly dependent on the advanced and expensive metallic instruments that are capable of serving in these extremely low-temperature and high-pressure environments. However, according to reports on metallic instruments serving in the offshore area, microorganisms play an important role on metal failure, which is called microbiologically influenced corrosion (Remazeilles et al., 2010; Zhao et al., 2018; Zhou et al., 2018; Ma et al., 2020). It was found that various bacteria showed different effects on metal failure process, i.e., corrosion acceleration, corrosion inhibition, or irrelevance—for example, sulfate-reducing bacteria are recognized as the corrosion-accelerating bacteria (Enning and Garrelfs, 2014; Guan et al., 2020), while some metal-reducing bacteria have been proven to successfully inhibit metal corrosion (Zuo, 2007). However, in a real marine environment, especially in the little-known Yap Trench environment, the biofilms formed on metallic surfaces are complex, heterogeneous, and far more than the reported sulfate-reducing bacteria and metal-reducing bacteria (Li et al., 2017; Zhang et al., 2019a).

Previous studies revealed that dramatic differences showed up between the communities in surrounding seawater and the biofilms on various metallic surfaces. Thus, the microbial diversity in seawater and sediments provide us limited knowledge on analyzing the feasibility and predicting the failure of metallic instruments. Until now, nothing about the influence of hadal communities on these metallic materials is known. Hence, clarifying the microbial compositions on metallic surfaces makes a significant sense to predict the safety and service life of metallic instruments applied in Yap Trench.

What is more, the biofilms formed on metallic surfaces are not only highly dependent on the environment but also closely related to metal-inherent qualities, such as element components, alloy phases, and so on (Dang and Lovell, 2016). These inherent qualities make metal alloys display various surface status, including surface free energy, roughness, hydrophilic/lipophilic property, and electrostatic charge, which attract certain microorganisms to adhere—for example, no electronically charged surface was more attractive to marine *Pseudomonas* sp. rather than the hydrophilic and negatively charged surface (Fletcher and Loeb, 1979). It was also proved that diverse microbial communities develop on the surfaces of metallic plates, which differed from the surrounding oligotrophic bacteria in seawater (Li et al., 2017; Zhang et al., 2019a). Furthermore, marine surface-associated biofilms formed on the copper alloys possess distinct microbial compositions compared with those formed on aluminum alloys (Zhang et al., 2019b). As a result, figuring out the bacterial communities of the biofilm on metallic surfaces in hadal environments would greatly help researchers to evaluate the microbial influence on metallic instruments, which might be favorable to predict the failure process of these metallic instruments employed in deep-sea environments.

In this work, several typical metal alloys, including titanium alloy, aluminum alloy, and copper alloy, which are commonly used for deep-sea instruments were employed as testing substrates. These alloys were exposed in Yap Trench for 1 year to observe the bacterial communities of the formed biofilm. According to this research, an initial attempt is made to know more about hadal environments and concerned more on metallic instruments serving in Yap Trench.

## Experimental

### Sample Collection

Titanium alloy TA2, aluminum alloy ZAL, aluminum alloy 5A06, aluminum alloy 1060, copper alloy T2, copper alloy B10, and copper alloy B30 were employed in this study. The composition of the alloys is shown in Tables 1–3. Coupons of 40 mm × 120 mm × 5 mm, made of alloy TA2, alloy ZAL, alloy 5A06, alloy 1060, alloy T2, alloy B10, and alloy B30, were prepared separately for seawater immersion tests. The coupons were fixed in an insulated frame cage which was fastened on a subsurface buoy. This buoy was exposed in Yap Trench (138°43′434″ E, 9°51′0215″ N) at 5,772 m undersea. The salinity of the seawater was detected as 3.47%, and the temperature was 1.58°C. The pressure at the exposure location was determined to be 5,881 MPa.

**TABLE 1:** Chemical composition of the alloys.

	Alloy TA2	Alloy ZAL	Alloy 5A06	Alloy 1060	Alloy T2	Alloy B10	Alloy B30
Ti (%)	Residual	0.15–0.35	0.02–0.10	≤0.03	/	/	/
Al (%)	/	Residual	Residual	Residual	/	/	/
Cu (%)	/	4.50–5.30	≤0.10	≤0.05	Residual	Residual	Residual
Fe (%)	≤0.30	≤1.00	0.00–0.40	/	≤0.005	≤0.02	≤0.90
C (%)	≤0.15	/	/	/	≤0.03	≤0.03	≤0.05
N (%)	≤0.05	/	/	/	/	/	/
O (%)	≤0.20	/	/	/	/	/	/
Mn (%)	/	0.60–1.00	0.50–0.8	≤0.03	/	/	≤1.20
Mg (%)	/	≤0.05	5.8–6.8	≤0.03	/	/	/
Si (%)	/	≤0.30	≤0.40	≤0.25	/	/	≤0.15
Zn (%)	/	≤0.20	≤0.20	≤0.05	/	/	/
V (%)	/	/	/	≤0.05	/	/	/
Ni (%)	/	≤0.10	/	/	/	/	/
Zr (%)	/	≤0.20	/	/	/	/	/
Ni–Co (%)	/	/	/	/	/	9.50–10.50	29.00–33.00
Pb (%)	/	/	/	/	≤0.005	≤0.01	≤0.05
S (%)	/	/	/	/	≤0.01	≤0.01	≤0.01
Bi (%)	/	/	/	/	≤0.02	≤0.02	/
Sb (%)	/	/	/	/	≤0.005	≤0.005	/
P (%)	/	/	/	/	≤0.01	≤0.01	≤0.006
As (%)	/	/	/	/	≤0.01	≤0.01	/

*The chemical element compositions (mass fraction%) of alloy TA2, alloy ZAL, alloy 5A06, alloy 1060, alloy T2, alloy B10, and alloy B30 employed in this research were according to national standards GB/T 3620.1-2016, GB/T 3190-2008, and GB/T 5231-2001.*

The immersion started from 23 May 2016 to 2 June 2017, which lasted for 375 days. After exposure, coupons were stored at −20°C until they were taken back to the laboratory.

A sterilized soft brush was used to scrape the biofilm from each coupon surface. Then, the biofilm was transferred into a sterilized beaker with phosphate-buffered saline. The biomass in solution was further filtrated through 0.22-μm filter membranes to obtain a concentrate of the microorganisms (Mittelman et al., 1997). These biofilm samples collected from alloy TA2, alloy ZAL, alloy 5A06, alloy 1060, alloy T2, alloy B10, and alloy B30 were named as TA2, ZAL, Al5A06, Al1060, T2, B10, and B30, respectively. Based on the differences of the major component in each metallic alloy, TA2, as a representative titanium alloy, was named as group A1; ZAL, Al5A06, and Al1060, as representatives of aluminum alloy, were named as group A2; and T2, B10, and B30, as representatives of copper alloy, were named as group A3.

### DNA Extraction and Sequencing

The DNA of these microorganisms was extracted using a previously reported method. DNA concentration and purity were determined with a spectrophotometer (Lambda 1A; Perkin-Elmer). A A260/A280 ratio of 1.8–2.1 was considered acceptable for PCR-based procedures (Zhang et al., 2019a). The extracted DNA of the biofilms was used as a template to amplify the 16S rRNA genes by PCR with the universal forward primer 338F (5′-ACTCCTACGGGAGGCAGCA-3′) and reverse primer 806R (5′-GGACTACHVGGGTWTCTAAT-3′). PCR purification kit (QIAGEN, Hilden, NRW, Germany) was used to purify the PCR products. The PCR libraries were conducted using TruSeq DNA PCRFree Sample Preparation Kit (Illumina, San Diego, CA, United States). After quantification with Qubit, the PCR libraries were sequenced on the Illumina HiSeq PE250 platform.

### Sequence Data Analysis

Based on the unique barcodes of each sample, raw paired-end reads were assigned. Subsequently, FLASH (V1.2.7) was used to merge these reads according to their overlap after the barcodes and primer cuts (Magoc and Salzberg, 2011). Then, based on the process for quality control in QIIME, these sequences were filtered, followed by detecting and removing the chimera sequences by UCHIME algorithm (Caporaso et al., 2010; Edgar et al., 2011). Operational taxonomic units (OTUs) were clustered with 97% similarity using UPARSE software, version 7.11 (Edgar, 2013). The taxonomy of each 16S rRNA gene sequence was analyzed by RDP Classifier 2.2 against the GreenGene database (DeSantis et al., 2006; Wang et al., 2007).

Then, the sequence data were normalized after unique tags dislodge to analyze OTU abundance and diversity index. Good coverage was calculated by QIIME to represent sequencing depth. Alpha diversity indices were employed to indicate the bacterial diversity of each sample, including Chao and ACE for species richness, Simpson and Shannon for community diversity evaluating both the species richness and evenness, and Faith’s phylogenetic diversity (Faith pd) for phylogenetic diversity. Besides these, beta diversity using clustering analysis and principal coordinate analysis based on unweighted unifrac distances were employed for the comparison of the community differences between groups. Furthermore, Venn diagrams were used to show the unique and shared OTUs of the three groups, and PICRUSt2 (Langille et al., 2013) was employed to predict the functional genes based on the 16S rRNA sequencing data, which were annotated against the Kyoto Encyclopedia of Genes and Genomes (KEGG) database V2018-01 (Kanehisa et al., 2017). Statistical tests based on analysis of variance were used to determine the difference in functional gene abundance, and factors with *p*-values less than 0.05 were considered to have a significant difference.

### Data Availability

The raw sequences obtained were deposited in the NCBI Short Read Archive database under Bioproject accession number PRJNA438021, with Biosample numbers SAMN23711893-23711899.

## Results

### Microbial Richness and Diversity of Biofilm on the Alloys

A total of 331,905 high-quality bacterial sequences, ranging from 38,102 to 58,460, were obtained for further analysis.

**FIGURE 1:**
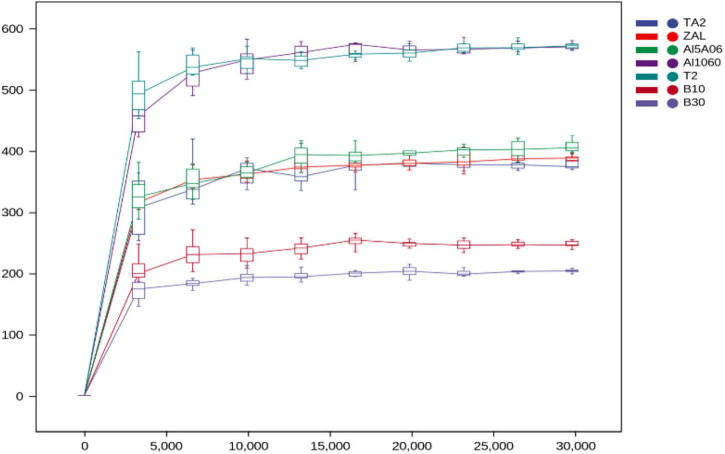
Rarefaction curves of the sequencing reads of the 16S rRNA genes from seven samples in Yap Trench for bacteria at 97% sequence similarity cutoff value (TA2, ZAL, Al5A06, Al1060, T2, B10, and B30 represented the biofilms collected from the corresponding alloy surfaces. The abscissa represented the extraction depth, and the ordinate represented the index and boxplot of the median alpha diversity after 10 calculations. The gentleness of the curve reflected the influence of sequencing depth on the diversity of these samples).

As shown in Table 2, the bacterial coverage ranged from 99.87 to 99.97%, indicating that the sequences obtained by V3–V4 Illumina sequencing captured their core microbial communities. In addition, all rarefaction curves of bacteria reached saturation, revealing that the amount of sequencing data was enough to capture the great majority of bacterial communities (Figure 1). The observed species and Chao1, which represented species richness of each sample, were quite different in these samples. In total, 556 species (Chao1 index 569 and ACE index 573) were found on titanium alloy TA2. On average, 274 observed species (Chao1 index 287 and ACE index 290) were found on aluminum alloys, and 432 observed species (Chao1 index 447 and ACE index 440) were on copper alloys. The biofilm on ZAL alloy showed the highest species richness among the three aluminum alloys, and the biofilm on T2 alloy showed the highest value among copper alloys, although the average values of the observed species, Chao1 index, and ACE index showed the following trend: titanium alloy > copper alloy > aluminum alloy. The Shannon and Simpson indices of these samples, revealing community diversities, showed similar results. The Shannon and Simpson indices of TA2 were 4.33 and 0.78, respectively. The average Shannon and Simpson indices of aluminum alloys were 4.80 and 0.91, while those of copper alloys were calculated as 4.98 and 0.89. Faith pd (shown in Table 2) was used to evaluate the evolution differences. The Faith pd indices of TA2 (57.10) and T2 (45.52) were obviously higher than the other samples, showing relatively high phylogenetic diversities.

**TABLE 2:** Diversity estimators for bacteria from seven metallic surface samples exposed in Yap Trench using 16S rRNA gene sequencing.

Sample	Observed species	Good coverage	Chao1	Faith’s phylogenetic diversity	ACE	Shannon	Simpson
TA2	556	0.9987	569	57.10	573	4.33	0.78
ZAL	383	0.9987	407	34.65	407	5.25	0.92
Al5A06	241	0.9994	248	28.42	258	4.34	0.88
Al1060	197	0.9997	205	19.15	205	4.82	0.92
T2	555	0.9988	573	45.52	565	5.67	0.91
B10	366	0.9989	380	38.15	379	5.01	0.92
B30	374	0.9990	389	34.56	375	4.26	0.84

*TA2, ZAL, Al5A06, Al1060, T2, B10, and B30 represented the biofilms collected from the corresponding alloy surfaces. The observed species, Chao1 and ACE, represented the species richness of each sample. The Shannon and Simpson indices of these samples revealed community diversities, and Faith’s phylogenetic diversity evaluated the evolution differences.*

### Comparison of the Microbial Composition of Biofilms on the Alloys

Differences in the composition of the bacterial community were detected for the seven alloys, i.e., A1 group (TA2), A2 group (ZAL, Al5A06, and Al1060), and A3 group (T2, B10, and B30).

**FIGURE 2:**
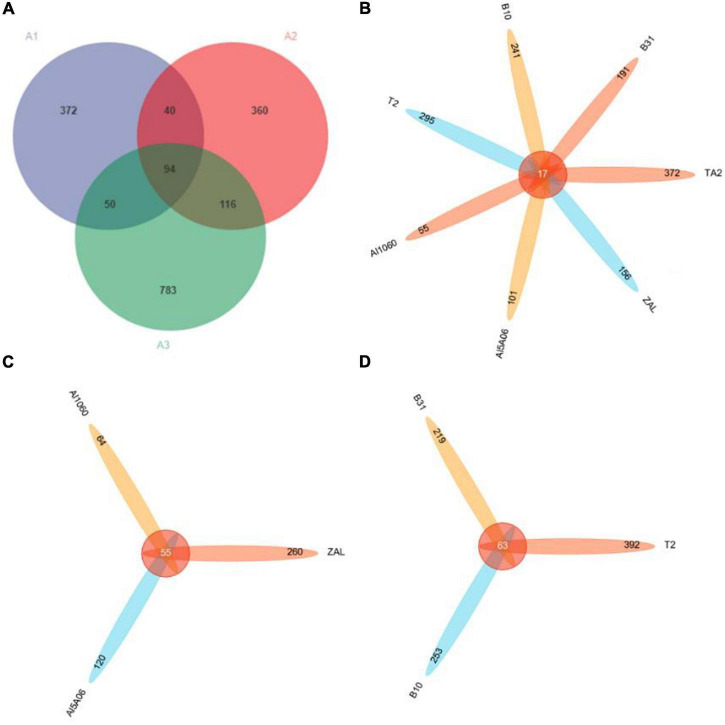
Venn diagrams of seven samples in Yap Trench based on 16S rRNA gene sequencing at a distance of 0.03. **(A)** Diagram of all samples at group level, **(B)** diagram of all samples at sample level, **(C)** diagram of aluminum alloys, **(D)** diagram of copper alloys (TA2, ZAL, Al5A06, Al1060, T2, B10, and B30 represented the biofilms collected from the corresponding alloy surfaces. A1 group referred to the titanium sample TA2; A2 group referred to the aluminum alloy samples ZAL, Al5A06, and Al1060; and A3 group referred to the copper alloy samples T2, B10, and B30).

As shown in Figure 2A, in the Venn diagram, there were 556 OTUs shown in group A1 (titanium alloy), 610 OTUs in group A2 (aluminum alloys), and 1,043 OTUs in group A3 (copper alloys). They shared only 94 OTUs at group level. The A2 and A3 groups shared more OTUs (116 OTUs) than those they shared with the A1 group (i.e., 40 OTUs for A1 and A2 groups and 50 OTUs for A1 and A3 groups). As to the sample level (Figure 2B), all seven samples only shared 17 OTUs, revealing dramatic differences in species composition. TA2 showed the highest unique OTUs of up to 67%, while Al1060 showed the lowest at 28%. Even in each group, the shared OTUs were found to be as low as 55 OTUs for the aluminum alloy samples (Figure 2C) and 63 OTUs for the copper alloy samples (Figure 2D).

**FIGURE 3:**
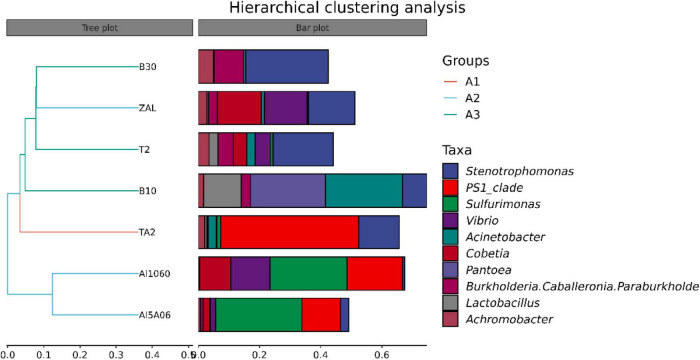
Hierarchical cluster tree of seven samples in Yap Trench using the average pair-group method based on the unweighted unifrac distance for bacteria (TA2, ZAL, Al5A06, Al1060, T2, B10, and B30 represented the biofilms collected from the corresponding alloy surfaces. A1 group referred to the titanium sample TA2; A2 group referred to the aluminum alloy samples ZAL, Al5A06, and Al1060; and A3 group referred to the copper alloy samples T2, B10, and B30. The panel on the left was a hierarchical clustering tree with the abscissa representing the unweighted unifrac distance. Samples were clustered according to their similarity. The panel on the right was a stacked histogram of the top 10 genera in abundance).

**FIGURE 4:**
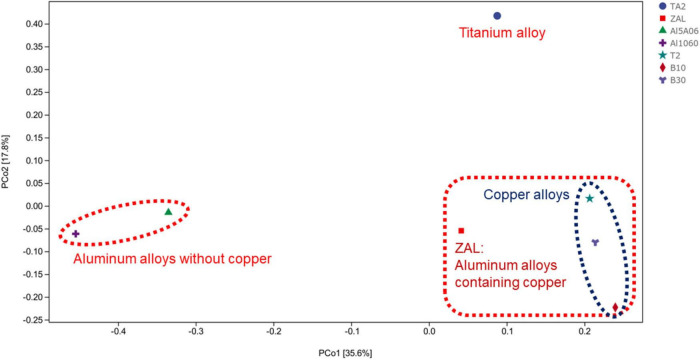
Principal coordinate analysis plots of unweighted unifrac between seven samples in Yap Trench based on operational taxonomic units using 16S rRNA gene sequencing (TA2, ZAL, Al5A06, Al1060, T2, B10, and B30 represented the biofilms collected from the corresponding alloy surfaces. The percentages in brackets on the axes represented the proportion of sample variance data that can be explained by the corresponding axes).

The average pair–group method with an arithmetic mean based on unweighted unifrac distances was performed to determine the differences between these samples, as shown in Figure 3. The clustering analysis showed that these samples could be divided into three groups: titanium alloy (TA2); copper alloys and aluminum alloy with copper element (T2, B10, B30, and ZAL); and aluminum alloys without copper (Al1060 and Al5A06). Furthermore, principal coordinate analysis (PCoA) plots of unweighted unifrac distances based on OTUs are shown in Figure 4. The results showed that different brands of each alloys were similar, such as the aluminum alloys without the copper group and the copper alloy group. An exception was found on ZAL, a kind of aluminum alloy containing copper, which was similar to copper alloys instead of aluminum alloys.

### Bacterial Community Compositions of Biofilms on the Alloys

**FIGURE 5:**
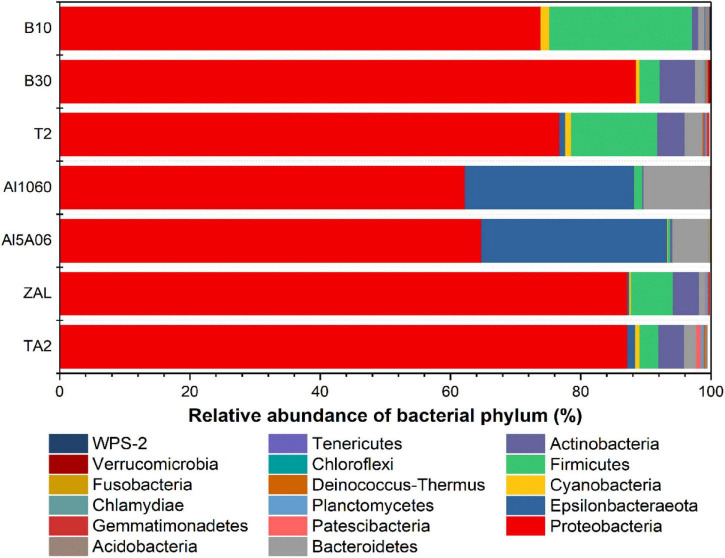
Relative abundance of 16S rRNA gene sequences of the seven samples exposed in Yap Trench for 1 year at the bacterial phylum level (TA2, ZAL, Al5A06, Al1060, T2, B10, and B30 represented the biofilms collected from the corresponding alloy surfaces. The top 10 abundant phyla of each sample are shown in this figure).

In total, more than 21 bacterial phyla were found in these samples. In terms of the average abundance of seven samples, Proteobacteria was found to be the dominant phylum, accounting for 77% of the total sequences (Figure 5). Then, it was followed by Epsilonbacteraeota accounting for 8%, Firmicutes accounting for 7%, Bacteroidetes accounting for 3%, and Actinobacteria accounting for 3%. Cyanobacteria, Acidobacteria, Planctomycetes, and Patescibacteria were also found in the samples with a relatively low proportion.

Corresponding to the hierarchical cluster tree and PCoA plot results, Al5A06 and Al1060 showed similar community compositions, while T2, B10, B30, and ZAL clustered more closely with each other in general. At the phylum level (Figure 5), Proteobacteria was the dominant phylum in all samples, ranging from 62 to 89%. Firmicutes were found as the second represented phylum on titanium alloy TA2 and copper-present alloys B10, B30, T2, and ZAL. However, Epsilonbacteraeota was the secondary represented phylum on non-copper aluminum alloys Al1060 and Al5A06. On the class level, Alphaproteobacteria (58%) was the dominant class on titanium alloy TA2, while Gammaproteobacteria (40–65%) was the dominant class on the copper alloys and aluminum alloys. Gammaproteobacteria (28%) took the second place, followed by Actinobacteria (3%), Bacteroidia (2%), Bacilli (2%), Campylobacteria (1%), and Deltaproteobacteria (1%) on TA2. Furthermore, Alphaproteobacteria (19–40%) took the second place on copper alloys and aluminum alloys except for B10, on which Bacilli was found to be the secondary (Table 3).

**TABLE 3:** Relative abundance of 16S rRNA gene sequences of the seven samples exposed in Yap Trench for 1 year at the bacterial class level.

Sample	TA2	ZAL	Al5A06	Al1060	T2	B10	B30
Alphaproteobacteria	58%	22%	19%	21%	29%	9%	40%
Gammaproteobacteria	28%	65%	41%	41%	47%	65%	48%
Actinobacteria	3%	3%	0%	0%	4%	1%	5%
Bacteroidia	2%	1%	6%	10%	3%	1%	1%
Bacilli	2%	5%	0%	1%	6%	20%	1%
Campylobacteria	1%	0%	29%	26%	1%	–	0%
Deltaproteobacteria	1%	0%	5%	1%	1%	0%	0%
Clostridia	1%	1%	0%	–	55%	2%	1%

*TA2, ZAL, Al5A06, Al1060, T2, B10, and B30 represented the biofilms collected from the corresponding alloy surfaces. The top five abundant classes of each sample are shown in this table.*

**FIGURE 6:**
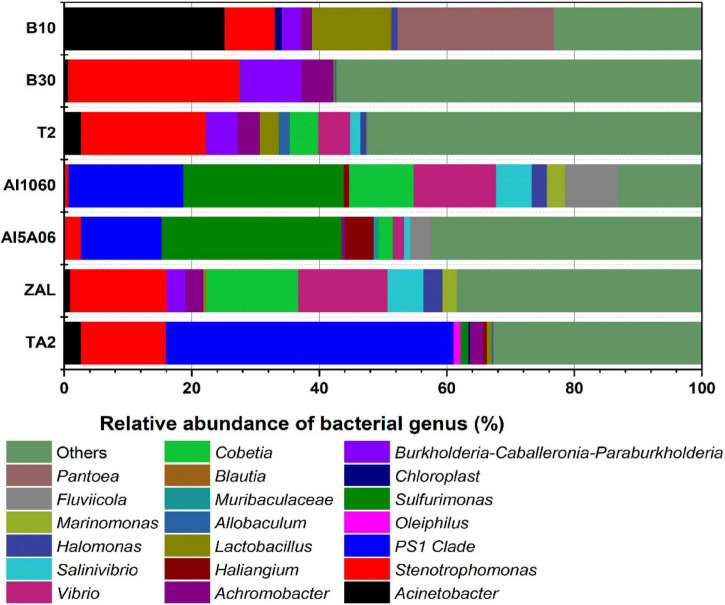
Relative abundance of 16S rRNA gene sequences of the samples exposed in Yap Trench for 1 year at the bacterial genus level (TA2, ZAL, Al5A06, Al1060, T2, B10, and B30 represented the biofilms collected from the corresponding alloy surfaces. The top 10 abundant genera of each sample are shown in this figure).

However, distinct dominant bacteria were found on different metals at the genus level (Figure 6). *PS1 Clade* (45%), *Stenotrophomonas* (13%), and *Acinetobacter* (3%) made up the dominant genus on TA2. *Sulfurimonas* (27% on average) and *PS1 Clade* (15% on average) were dominant on non-copper aluminum alloys Al1060 and Al5A06, while *Stenotrophomonas* (15%), *Cobetia* (14%), and *Vibrio* (14%) were dominant on the copper-present aluminum alloy ZAL. *Stenotrophomonas* (18% on average), *Burkholderia*–*Caballeronia*–*Paraburkholderia* (6% on average) and *Achromobacter* (3% on average) formed the communities on copper alloys B10, B30, and T2. These results illustrated that the composition of microbial communities attached on the metal surfaces highly depended on the metal composition.

### Key Functional Gene Prediction

**FIGURE 7:**
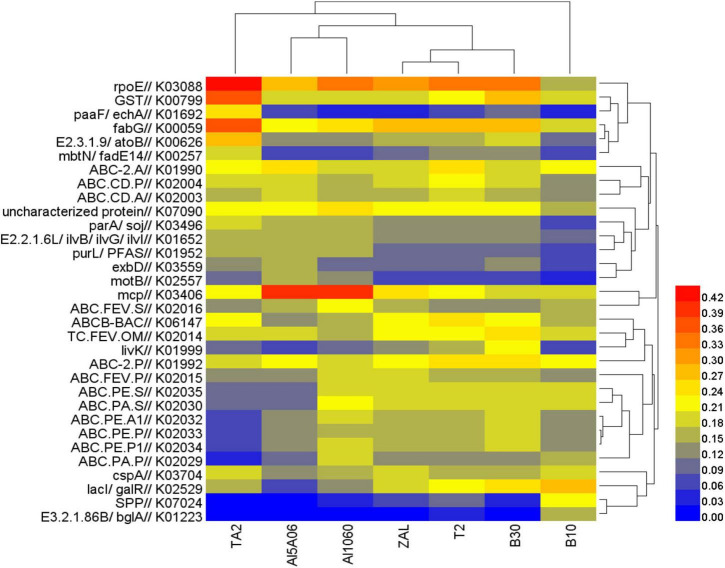
Heat map of functional genes assigned with Kyoto Encyclopedia of Genes and Genomes database in the seven samples in Yap Trench using 16S rRNA gene sequencing (TA2, ZAL, Al5A06, Al1060, T2, B10, and B30 represented the biofilms collected from the corresponding alloy surfaces. The value of the color scale represents the relative abundance of functional genes).

The functional gene profile of the microbial communities on the seven samples was analyzed by PICRUSt2 based on KEGG database. Figure 7 shows the relative abundance of the top 15 identified genes in each sample. Distinctive functional genes were found in the biofilms on different metal alloys. The most abundant functional gene sets were RNA polymerase sigma-70 factor-encoding gene (*rpoE*) on TA2 (0.44%), Al5A06 (0.31%), Al1060 (0.33%), ZAL (0.31%), T2 (0.34%), B10 (0.18%), and B30 (0.35%). Besides this, glutathione S-transferase-encoding gene (*GST*), which played an important role in biodefense system, was shown to be relatively high in these samples. Another 3-oxoacyl-[acyl-carrier protein] reductase-encoding gene (*fabG*) showed a high abundance, which might be related to fatty acid synthesis and environmental tolerance. What is more, methyl-accepting chemotaxis protein-encoding gene (*mcp*) was found to be dramatically abundant on non-copper aluminum alloys Al1060 and Al5A06. The ABC-2-type transport system permease protein-encoding gene (*ABC-2.P*), LacI family transcriptional regulator-encoding gene (*lacI* and *galR*), and ATP-binding cassette-encoding gene (*ABCB-BAC*) were found to be relatively rich on copper-containing alloys ZAL, T2, B30 and B10.

### Copper Resistance Genes

Since copper is considered toxic to microorganisms, various genes related to copper resistance, such as copper tolerance two-component regulatory system *cusSR*, Cu^+^ transporting ATPase-encoding genes *copAB* (Silver and Phung, 2005), and copper resistance protein-encoding genes *pcoBCD*, were identified in these samples and shown in Table 4. According to the copper contents in the alloys, these samples could be divided into two groups, i.e., copper-free alloys (TA2, Al1060, and Al5A06) and copper-present alloys (ZAL, T2, B10, and B30). It was found that the abundance of *cusRS* genes of copper-free alloys was significantly lower than that of copper-present alloys (*P* < 0.01, Student’s *t*-test), while the abundance of *copAB* genes of copper-free alloys was higher than that of copper-present alloys, which showed a significant difference (*P* < 0.01, Student’s *t*-test). Besides these, most of the *pcoBCD* genes were shown to be more abundant in copper-present alloys than in copper-free alloys with *P* < 0.01 (for *pcoBC* genes).

**TABLE 4:** Relative abundance of key functional genes related to copper resistance in the seven samples in Yap Trench using 16S rRNA gene sequencing.

Gene	Description	TA2	ZAL	Al5A06	Al1060	T2	B10	B30
*cusR*	Copper resistance phosphate regulon response regulator	0.009%	0.021%	0.004%	0.005%	0.022%	0.027%	0.029%
*cusS*	Heavy metal sensor histidine kinase	0.010%	0.021%	0.004%	0.006%	0.023%	0.027%	0.030%
*copA*	Cu^+^-transporting ATPase	0.066%	0.051%	0.091%	0.096%	0.053%	0.051%	0.044%
*copB*	Cu^+^- transporting ATPase	0.054%	0.033%	0.047%	0.044%	0.031%	0.019%	0.029%
*pcoB*	Copper resistance protein B	0.014%	0.022%	0.004%	0.005%	0.026%	0.021%	0.030%
*pcoC*	Copper resistance protein C	0.012%	0.023%	0.004%	0.006%	0.024%	0.026%	0.029%
*pcoD*	Copper resistance protein D	0.013%	0.023%	0.004%	0.006%	0.022%	0.025%	0.026%

*TA2, ZAL, Al5A06, Al1060, T2, B10, and B30 represented the biofilms collected from the corresponding alloys surfaces.*

## Discussion

Yap Trench has attracted much attention due to its specific physical and geochemical characteristics as well as its hadal biosphere. Several studies have reported the microbial diversity and metabolic potentials of seawater and surface sediment (Zhang et al., 2018; Fu et al., 2020), but seldom focused on the microbial composition on the serving metals in Yap Trench. This research provided a brief glimpse of the biofilm on several metal alloys at 5,772 m undersea in Yap Trench. The biofilms formed on these metals with distinct composition not only reflected the deep-sea environment to some degree but also provoked new thoughts of the interaction of microorganisms with metals in deep-sea conditions.

### Microbial Richness and Diversity Analysis

Metal alloy surfaces are ideal sites for biofilm formation and allowing biofilm-associated microorganisms to improve their growth (Beveridge et al., 1997; Loto, 2017). However, microbial richness and diversity showed obvious differences between the various alloy surfaces studied in this research. The highest richness was found on titanium alloy TA2 due to its best biocompatibility (Long and Rack, 1998; Niinomi, 2008; Geetha et al., 2009), followed by copper-present alloys (ZAL, T2, B10, and B30), and the least was aluminum alloys Al5A06 and Al1060. However, these results differed from previous shallow-sea results. Samples immersed at a depth of 1–1.5 m below sea level for 30 months in the coastal zone of Hongtang Bay showed that higher richness was found on aluminum alloy, while lower richness was found on copper alloy (Zhang et al., 2019a), which could be attributed to the oligotrophic environment, leading to the different planktonic microorganisms in Yap Trench. Gammaproteobacteria constituted up to 92.2% of the total microbial community in the hadal seawater of Yap Trench (Zhang et al., 2018), while Alphaproteobacteria (35.3%) made up the major bacterial groups in the shallow surface seawater (1–1.5 m below sea level) of Hongtang Bay (Zhang et al., 2019a). As a result, on the surface of copper alloys in the hadal environment, biofilms tended to be formed, which provided suitable living environments for microbial organisms (Zhang et al., 2019b). Besides this, an oxidation passivation film composed of Al_2_O_3_ usually formed on aluminum alloys (Wolowik et al., 1998). The super-hydrophobic property and oligotrophy might also influence the bacterial attachment (Yu et al., 2014; Xiao et al., 2015). Furthermore, the detection of various metal resistance genes, including Cu resistance, in the Yap Trench metagenomes was reported (Zhang et al., 2018), illustrating that hadal microorganisms would be more adapted to Cu-rich environments than to shallow seawater. The environmental copper-resistant microbiological composition as well as the surface conditions of the alloys both contributed to the high bacterial richness on copper-present alloys.

Among these seven alloys, even on the same type of alloys, such as copper alloys T2, B10, and B30, the composition of the bacterial communities showed great distinctions. The proportions of the unique OTUs of each copper alloy sample ranged from 78 to 86%, revealing that even alloying elements with low concentrations would greatly influence the bacterial communities.

### Microbial Community Analysis

Metallic surfaces might promote bacterial attachment and biofilm formation by enriching nutrients or acting as electron donors for microorganisms (Beveridge et al., 1997; Yu et al., 2013; Guan et al., 2016, 2021). Diverse and distinct bacterial communities developed on the surfaces of different alloys, which highly depended on the composition of the substrate.

On titanium alloy TA2, *PS1 Clade* played the leading role in the biofilm. *PS1 Clade* of Alphaproteobacteria was firstly isolated from a coastal station in the East Sea, Western Pacific Ocean, and reported by SJ Yang (Yang et al., 2012). *PS1 Clade* was a member of a putatively novel order closely related to Rhizobiales. The *PS1* lineage stem would adapt to various marine habitats, including the oligotrophic Sargasso Sea as well as tropical and temperate environments (Jimenez-Infante et al., 2014). The core genome of the *PS1 Clade* suggested an aerobic, heterotrophic lifestyle with genes encoding for gluconeogenesis, citric acid cycle, and the Entner–Doudoroff pathway, implying that the *PS1 Clade* might not be primary cellulose degraders but opportunists utilizing cellobiose and small oligosaccharides (Jimenez-Infante et al., 2014; Daniel and Ana, 2020). What is more important is that the genome of *PS1 Clade* strains represented numerous high-affinity transporter-encoding genes, which were genomic hallmarks for cells proliferating in low-nutrient environments (Lauro et al., 2009). *Stenotrophomonas* took the second place in TA2 biofilm. Although it was well-known as a nosocomial and human infection pathogen (Coenye et al., 2004), *Stenotrophomonas* strains dwelling in marine environments remained unclear. Many *Stenotrophomonas* strains showed high resistance to high-level intrinsic resistance to heavy metals. It was also proved that they could degrade a wide range of organic compounds, including pollutants, which would potentially be used in bioremediation (Ryan et al., 2009). The metabolites of *Stenotrophomonas* strains always showed antifungal or antibacterial activities (Romanenko et al., 2008), which led to a biofilm with a relatively simple constitution on the TA2 surface. Another dominating genus, *Acinetobacter* on TA2, is one of the commonly found Gram-negative bacteria in marine environments. Some *Acinetobacter* species isolated from deep-sea sediments were found to be cold-adapted (Xue et al., 2019). *Acinetobacter* played an important role in hydrocarbon degradation and has a key role in bioremediation processes (MacCormack and Fraile, 1997). Various *Acinetobacter* strains were reported to be oil-, sulfonamide-, and phenol-degrading (Kobayashi et al., 2012; Zhang et al., 2012; Luo et al., 2013), indicating that they could make use of multiple organic carbon sources. Thus, *Stenotrophomonas* and *Acinetobacter* might act as the primary degraders for *PS1 Clade*, forming mutualistic symbiosis in the biofilm. It is worth mentioning that although the hadal environment was considered oligotrophic, organic matters were discovered in this area, and heterotrophic processes were found as the most common microbial metabolisms in the Yap Trench seawater (Zhang et al., 2018). Researchers took the view that the typical “V-shape” topography of the trenches would accumulate organic matters by a funneling effect. These organic matters could come from sinking particulates from the upper ocean, terrestrial inputs, chemosynthesis from the dark ocean, or even cell lysates at the trench axis (Jover et al., 2014). Then, under gravity, these organic materials would slowly migrate to the deepest trench axis (Ichino et al., 2015). The funneling effect due to the “V-shape” of the trench flanks also played an important role in the formation of increasing surface sedimentary organic carbon content which might come from the upper seawater layer (Li D. et al., 2020). Besides these, abundant genes involved in the degradation of various types of carbohydrates, hydrocarbons, and aromatics were reported by previous studies (Zhang et al., 2018), showing their potentials to be used organic carbon sources in the Yap Trench environment indicating organic matter-enriched environments.

On non-copper aluminum alloys Al1060 and Al5A06, *Sulfurimonas* and *PS1 Clade* dominated the bacterial groups in the biofilms. The genus *Sulfurimonas* belonged to the class Campylobacteria within the phylum of Epsilonbacteraeota (Inagaki et al., 2003). *Sulfurimonas* strains were discovered in various habitats, including marine sediments, deep-sea hydrothermal vents, and pelagic water column redoxclines (Han and Perner, 2015). Although the lineage *Sulfurimonas* was well-known as small sulfur-oxidizing bacteria utilizing reduced sulfur compounds such as sulfide, thiosulfate, and elemental sulfur as an electron donor for growth, organic compounds including formate, fumarate, amino acid, and alcohol mix could work as a preferred electron donor and contribute to bacterial growth (Labrenz et al., 2013). The versatile metabolic strategies of *Sulfurimonas* species helped them adapt to a broad type of environments (Han and Perner, 2015), including the deep-sea environment in Yap Trench. The versatile metabolic strategies might also cooperate with *PS1 Clade* to form mutualistic symbiosis in the biofilm.

On copper alloys T2, B10, 30, and copper-present aluminum alloy ZAL, *Stenotrophomonas* was found as a major constitution in these biofilms. As mentioned above, *Stenotrophomonas*, which phylogenetically belonged to Gammaproteobacteria, showed high resistance to heavy metals, including Cu (Ye et al., 2013; Chen et al., 2016; Hou et al., 2020). As a result, the *Stenotrophomonas* strains were successfully isolated from various copper-rich environments, such as copper-polluted agricultural soils and well-adapted Cu(II)-reduced biocathodes of microbial fuel cells (Altimira et al., 2012; Tao et al., 2017). *Stenotrophomonas* strains could convert Cu(II) into Cu(0) on the cell surface in the absence of cathodic electrons (Shen et al., 2017). On copper surface, *Stenotrophomonas* tended to release more amounts of extracellular polymeric substances (EPS) to form biofilms with a strong Cu(II) complexation effect (Hou et al., 2020). Therefore, *Stenotrophomonas* showed high tolerance or might get use of Cu(II) on copper alloy surfaces by forming a biofilm with high EPS. At the same time, this Cu(II) reduction process would inhibit the corrosion of the copper alloys serving in this environment because the essence of corrosion was defined as the oxidation process of metals. Due to the existence of a biofilm composed of *Stenotrophomonas* which would reduce Cu(II) (Shen et al., 2017), the oxidation process was restrained, leading to an inhibited corrosion process. *Burkholderia–Caballeronia–Paraburkholderia* showed high proportions on these copper-present alloys, too. They were discovered on copper-rich microbial fuel cells, revealing a high tolerance to copper (Wu et al., 2019; Ai et al., 2020). Another dominating genus in the biofilm on B10 was *Acinetobacter*. Besides the cold-adapting and hydrocarbon-degrading characteristics mentioned above, biosorption of Cu(II) was found on *Acinetobacter* in 2017 (Zhang et al., 2017). In the biofilm containing *Acinetobacter*, Cu(II) would promote protein secretion and bound with EPS, thus leading to more compact granules with better ability to settle (Jiang et al., 2020). Above all, the biofilm formed on copper-present alloys all showed high tolerance to copper. The biofilm would use, reduce, or biosorpt Cu(II) to form a stable and functional mutualistic symbiosis.

Besides that, previous reports showed that the abundant genes of bacterial communities in Yap Trench seawater were involved in stress response and metal resistance (Zhang et al., 2018). The attached bacteria on these alloys were considered to be derived from the planktonic communities and enriched on metal surfaces. Thus, high bacterial diversity and stable functional mutualistic symbiosis could form on metallic surfaces, even on toxic copper alloys.

### Key Functional Gene Analysis

The functional gene *rpoE* was found to be relatively abundant in all seven samples. *RpoE* gene was known as an important stress response gene. *RpoE* could encode key RNA polymerase component, which contributed to the protein expression for periplasmic and outer membrane component integrity (Helmann, 2002; Woods and McBride, 2017). The relatively high abundance of *rpoE* revealed a high resistance of these biofilms to the extreme deep-sea environment with low temperature, high pressure, and oligotrophic features. *FabG* gene encoding 3-oxoacyl-[ACP] reductase and *GST* gene encoding glutathione S-transferase were also commonly found in these samples. *FabG* gene was a key enzyme in the type II fatty acid synthase system in bacteria and catalyzes beta-ketoacyl-ACP reduction, while A played key roles on fatty acid biosynthesis (Li et al., 2006; Huang et al., 2008). The bacterial *GST*s were reported to be active in catalyzing specific reactions in the degradation pathways of recalcitrant chemicals for growth (Vuilleumier, 1997). Thus, the obvious presence of *fabG* and *GST* genes indicated harsh living conditions in Yap Trench (Vuilleumier and Pagni, 2002). Besides that, several genes connected to transport systems, such as *ABC.CD.P*, *ABC.CD.A*, *ABC-2.A*, *ABC-2.P*, and so on, were detected. ATP-binding cassette (*ABC*) transporter-encoding genes might mainly come from *PS 1 Clade*. *ABC* transporters were known to transport a wide variety of substrates, such as amino acids, oligopeptides, and sugars (Davidson et al., 2008). These *ABC* transporters contributed significantly to the uptake of extensive substrates for growth in relatively oligotrophic and pelagic environments.

It is worth mentioning that the existence of Cu element seemed to make a great influence on the functional genes of the biofilms than the other alloying elements. The abundance of several genes related to copper resistance, such as the copper tolerance two-component regulatory system *cusSR*, Cu^+^-transporting ATPase-encoding gene *copAB* (Silver and Phung, 2005), and copper resistance protein-encoding gene *pcoBCD*, was found to be distinctly differentiated. Based on copper contents, the alloys could be divided into two groups: copper-free alloys (TA2, Al1060, and Al5A06) and copper-present alloys (ZAL, T2, B10, and B30). The copper-free alloys showed low *cusSR* and *pcoBCD* but high *copAB* abundance, while the copper-present alloys revealed a contrast. The *cusS–cusR* two-component systems were significant for bacteria in sensing, responding, and adapting to the changing environments, such as the elevation of Cu(I) ions in the periplasm (Affandi and McEvoy, 2019). The plasmid-encoded gene *pcoBCD* would detoxify copper in the periplasm and further strengthen the copper resistance ability (Rensing and Grass, 2003), while *copA* and *copB* genes could encode Cu^+^-transporting ATPase, which would act as ATPase membrane pump to transport copper ions (Mana-Capelli et al., 2003; Silver and Phung, 2005). The high abundance of high *copAB* on copper-free alloys was an interesting phenomenon because of the opposite results compared to that obtained in shallow surface seawater (Zhang et al., 2019a,b). This might be attributed to the fact that, in the hadal environment, the environment might be quite oligotrophic with low copper contents (less than 0.000002% in surface sediments; Huang et al., 2020). No results were found in hadal seawater in Yap Trench; it might be even lower. However, copper was considered as one of the essential elements for living cells (Favre et al., 2019), so they needed to transport copper into the cell to enrich copper for growth and metabolism, which could lead to high *copAB*. That might also be the reason why the abundance of bacteria that survived on copper-free alloys was lower than that on copper alloys. So, based on these above-mentioned results, a hypothesis could be proposed. On copper-free alloys, the biofilms showed low copper-resistant gene abundance but high copper-sensitive gene abundance, which might be used for transporting copper ions according to the growth and metabolism requirements. On the contrary, the biofilms on copper-present alloys showed a relatively high copper resistance. They might not consume ATP to export copper ions. They might either make use of copper ions in the cell or export copper ions in other ways. The diverse response to copper led to totally different bacterial communities and functions of the biofilm on these alloys.

## Conclusion

This study caught a brief glimpse of biofilms formed on metal alloys in Yap Trench. Although it was known that planktonic bacterial communities showed a great difference with the biofilm communities, this research found out that the bacterial communities on the biofilms at various substrates revealed obvious differences. Among the alloys studied in this research, copper element showed strong influences on microbial communities and key functional genes even at a relatively low content in the alloy, such as ZAL. Titanium alloy provided a suitable substrate for a mutualistic symbiotic biofilm. Aluminum alloys without copper components showed the least bacterial richness and formed cold-adapted and oligotrophic-adapted biofilms. Copper-present alloys showed a relatively high bacterial richness with copper-resistant or even copper-utilizing biofilms on the surfaces. Besides that, the copper-related biofilm would participate in Cu(II) reduction, which could effectively inhibit copper corrosion. Furthermore, the bacterial communities of biofilms on these alloys were found to be highly different from those in shallow sea, and many bacterial genera remained unclear based on our existing database. Thus, future research on extreme environments, such as deep-sea environments, are critically needed and of great significance.

## Data Availability Statement

The datasets presented in this study can be found in online repositories. The names of the repository/repositories and accession number(s) can be found below: https://www.ncbi.nlm.nih.gov/, PRJNA438021 SAMN23711893-23711899.

## Author Contributions

XZ and WC were responsible for designing and conducting the experiments, analyzing the data, and drafting the manuscript. XZ and YZ revised the manuscript and provided much needed insight into the result interpretation. WC and PJ took part in plate placement and sample collection. JD have overseen all aspects of this project in terms of scientific significance. All authors contributed to the article and approved the submitted version.

## Conflict of Interest

The authors declare that the research was conducted in the absence of any commercial or financial relationships that could be construed as a potential conflict of interest.

## Publisher’s Note

All claims expressed in this article are solely those of the authors and do not necessarily represent those of their affiliated organizations, or those of the publisher, the editors and the reviewers. Any product that may be evaluated in this article, or claim that may be made by its manufacturer, is not guaranteed or endorsed by the publisher.
